# Data of antihyperlipidaemic activity for methanolic extract of *Tagetes patula* Linn. flower head along with piperine, as bioavailability enhancer

**DOI:** 10.1016/j.dib.2018.10.022

**Published:** 2018-10-13

**Authors:** Sneha Nawale, K. Padma Priya, P. Pranusha, M. Ganga Raju

**Affiliations:** a Department of Pharmacognosy, Gokaraju Rangaraju College of Pharmacy, Bachupally, Hyderabad 500090, India; b Gokaraju Rangaraju College of Pharmacy, Bachupally, Hyderabad 500090, India; c Department of Pharmacology, Gokaraju Rangaraju College of Pharmacy, Bachupally, Hyderabad 500090, India

**Keywords:** METP, Methanolic extract of *Tagetes patula* flower heads, GC–MS, gas chromatography and mass spectrometry, NO, nitric oxide, IAEC, Institutional Animal Ethical Committee, CPCSEA, Committee for the purpose of control and supervision of experimentation on animals, OECD, Economic Cooperation and Development, *i.p*, intraperitoneal, *p.o*, per oral, PTU, propylthiouracil, ANOVA, Analysis of variance, ROS, Reactive oxygen species, b. wt, body weight, TC, total cholesterol, TG, triglycerides, HDL, high density lipoprotein, LDL, low density lipoprotein, VLDL, very low density lipoprotein, SEM, standard error of mean, Antihyperlipidemic activity, *Tagetes patula*, Piperine, Triton X-100, Bioavailability, GC–MS

## Abstract

The data present in this article is associated with influence of piperine (secondary metabolite) on the antihyperlipidemic and antioxidant activity of methanolic extract of *Tagetes patula* (METP). METP was evaluated for antihyperlipidemic and antioxidant potential. Phytoconstituents of METP were identified using gas chromatography linked with a mass spectrometer. *in vivo* antihyperlipidemic activity of METP at the dose of 200 and 400 mg/kg b. wt. and 200 and 400 mg/kg b. wt. along with piperine (20 mg/kg b. wt.) were evaluated by Propylthiouracil induced and Triton X-100 induced hyperlipidemia in rats. Propylthiouracil significantly increased the serum TC (p<0.01), TG (p<0.01), LDL (p<0.01) and VLDL (p<0.01) levels and induction of HDL (p<0.01) at a dose of 400 mg/kg b. wt. along with piperine. Triton X-100 at a single dose of *i.p* increased lipid levels within 48 h. Increased lipid levels were significantly reduced TC (p<0.01), TG (p<0.01), LDL (p<0.05) and VLDL (p<0.05) by METP at doses of 200 and 400 mg/kg b. wt. along with piperine. Current data were also supported by histological study of livers, Cord pattern of hepatocytes, few periportal lymphocytes in focal area observed in hyperlipidemic rats and hepatocyte, periportal and centrilobular region of liver appear normal in treated group. METP along with piperine (capability to enhance bioavailability and has a property of increasing oral absorption of drugs) showed promising antioxidant and antihyperlipidemic activity which suggests the further use of *Tagetes patula* extract for the management of hyperlipidemia and atherosclerosis.

**Specifications table**

**Table:** 

**Subject area**	*Pharmacy*
**More specific subject area**	*Antihyperlipidemic activity of medicinal plant*
**Type of data**	*Table, text file, graph, figure*
**How data was acquired**	*Gas chromatography and mass spectroscopy was performed on*
	*Agilent 6890 series GC–MS instrument with HP-5MS Column*
	*(dimensions 30m^•^ ~0.32mm^•^ ~0.25μm) and semi auto analyser.*
**Data format**	*Analysed*
**Experimental features**	*Total cholesterol, triglyceride, HDL, LDL and VLDL was measured for METP(200 mg/kg bd.wt) METP(200 mg/kg bd.wt + piperine), METP(400 mg/kg bd.wt) and METP(400 mg/kg bd.wt + piperine)for triton induced and PTU induced hyperlipidaemia animal models.*
**Experimental factors**	*Methanol extract of flowers of the Tagetes patula was prepared By soxhlet extract assembly*
	1. *The acute toxicity data for methanol extract of flower heads.* 2. *Was performed by using female mice followed by OECD guidelines 425. Hyperlipidaemia was induced with propylthiouracil of 10 mg kg-*^*1*^ *b. wt. dosage and 0.01% PTU in drinking water for 7 days.* 3. *Rats were divided into seven groups of six rats (n=6) each. The Group I and II served as normal control and disease control, respectively receives saline (0.2 ml oral). Group III and IV, were treated with METP and Group V and VI were treated with METP along with piperine. The Group VII served as standard*
**Data source location**	*Department of Pharmacology, Gokaraju Rangaraju College of Pharmacy, Bachupally, Hyderabad-500090, Telanagana.*
**Data accessibility**	*All data are given along with the article and also provided in NCBI repository.*
**Related Research articles**	1. *Xin Di, Xin Wang, Xin Di, Youping. Effect of piperine on the bioavailability and pharmacokinetics of emodin in rats Linn. J Pharma Biomed Anal, 115(2015: 144-49).* 2. *Jabeen A., M. Ahmed, S. U. Simjee, Lubna,Samina B., S. Faizi.Anti-TNF-α and anti-arthritic effect of patuletin: A rare flavonoid from Tagetes patula. Int Immunopharmacol., 36 (2016): 232-40.* 3. *N. S. Adigun, A. T. Oladiji, T.O. Ajiboye. Antioxidant and anitihyperlipidaemic activity of hydroalcoholic seed extract of Aframomum melegueta K. Schum in triton X-100 induced hyperlipidemic rats. South Afri J Botany, 105(2016): 324-32.*

**Value of the data**

• The methods and data can be used to study *Tagetes patula* for its antihyperlipidaemic property studied in detail.
• Comparison of antihyperlipidaemic activity data of METP (200 and 400 mg/kg bd.wt.) alone and along with piperine (20 mg/kg bd.wt) as penetration enhancer also gives reference for researchers for formulation studies.
• GC–MS data and *in vitro* antioxidant activity data of METP also provide valuable reference to compare secondary metabolite and their action as antihyperlipidaemic activity. Furthermore Nutritional ingestion of this plant species will put in innovative scope in the managing of hyperlipidemia and other metabolic disorders.

## 1 Data

**Fig. 1:**
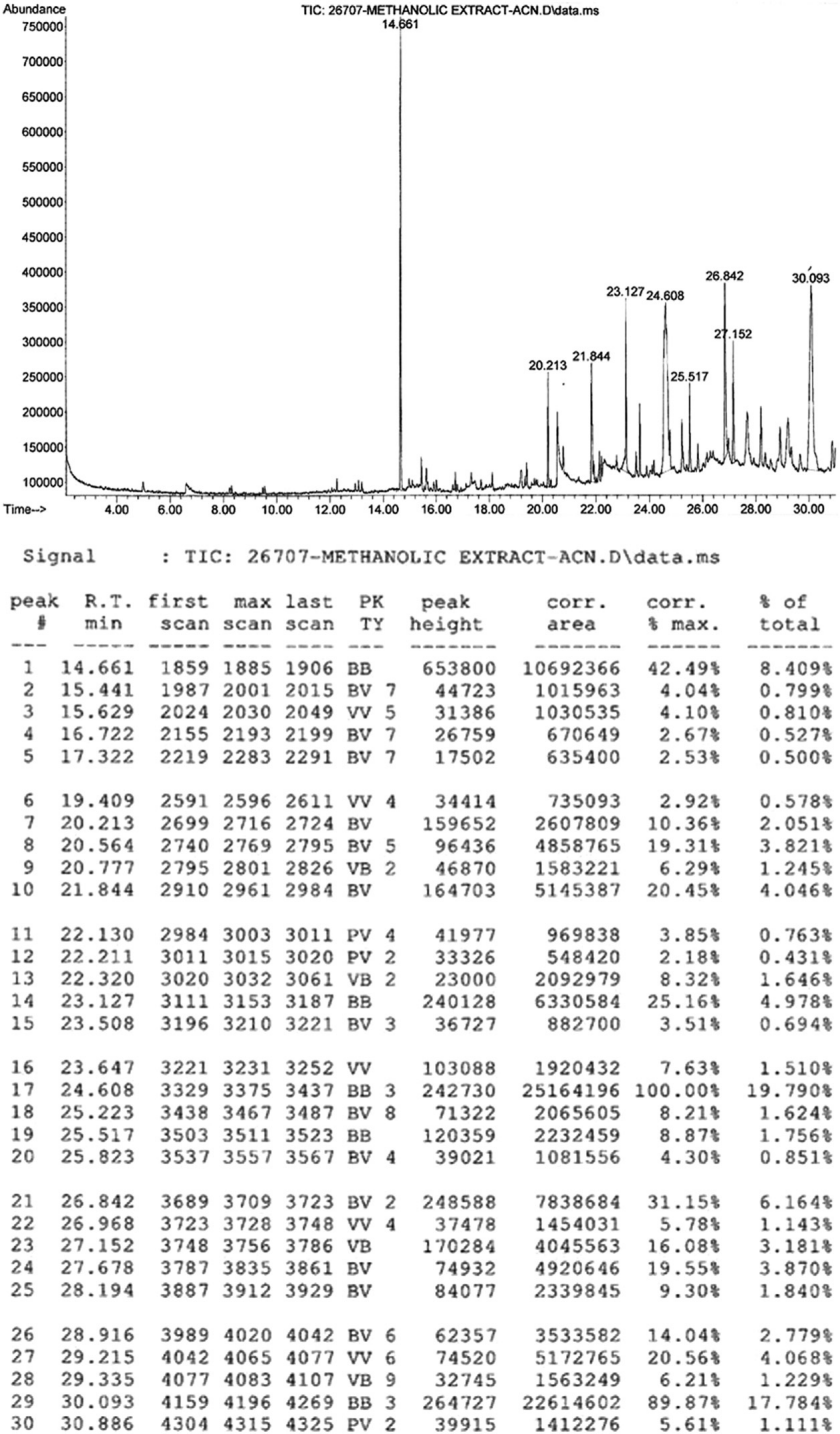
Gas chromatogram and mass spectrometry spectra of methyl extract of flower heads of *Tagetes patula* (METP).

**Fig. 2:**
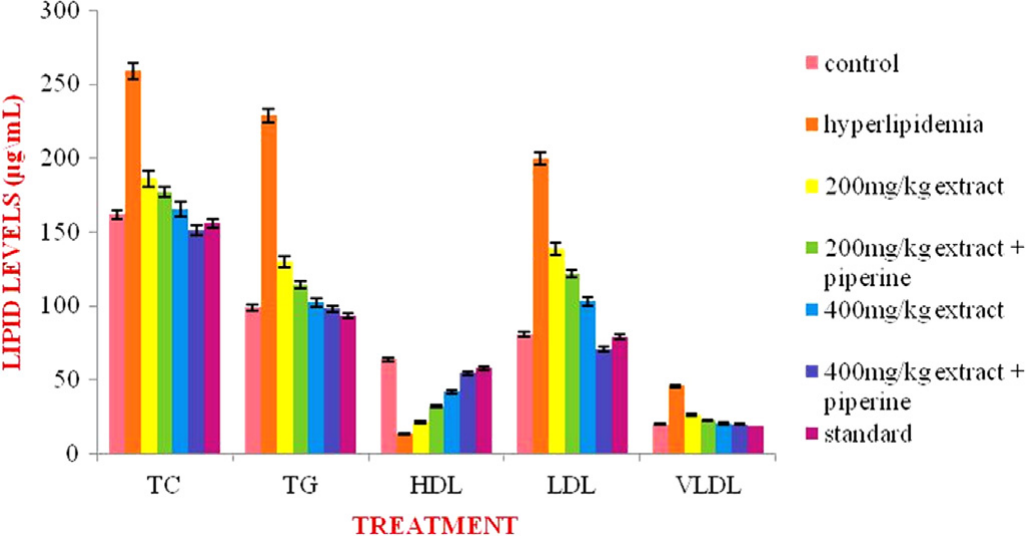
Effect of METP on lipid levels of propylthiouracil induced hyperlipidemia.

**Fig. 3:**
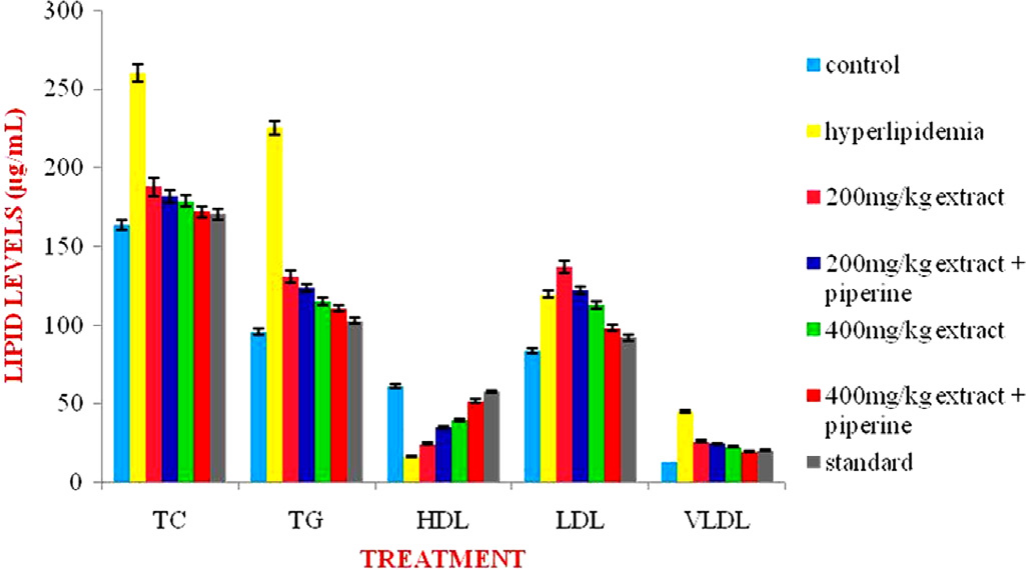
Effect of METP on lipid levels of Triton X-100 induced hyperlipidemia.

**Fig. 4:**
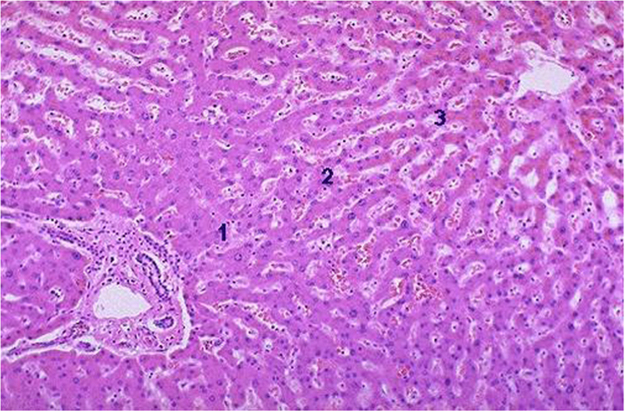
Histopathology of rat׳s liver in control group, Bile duct appeared normal, no inflammation or fibrosis noticed surrounding the portal region of liver. Kupffer cells and sinusoids are normal. No evidence of fatty change and fibrosis.

**Fig. 5:**
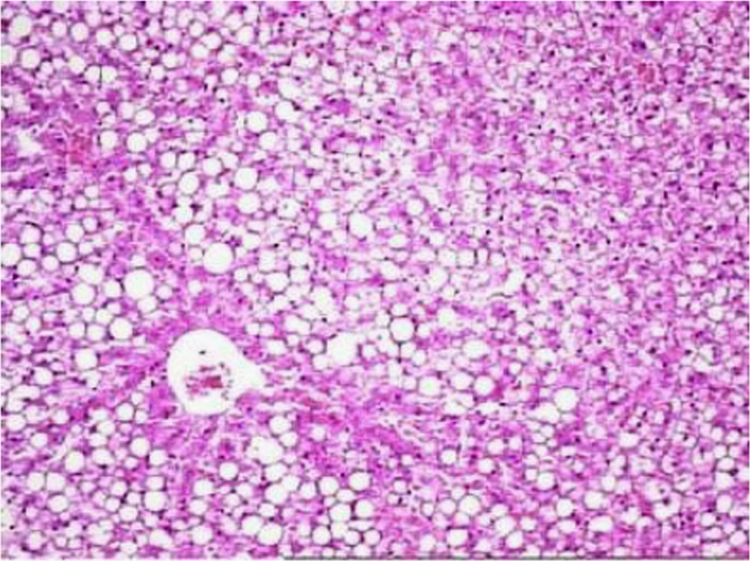
Cord pattern of hepatocytes. Few periportal lymphocytes in focal area fibrosis noticed in periportal region of liver. Fatty change found in cytoplasm and fibrosis.

**Fig. 6:**
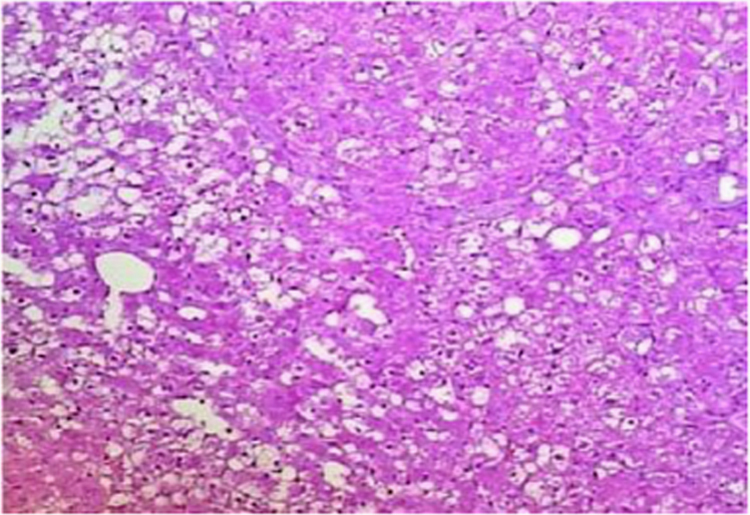
Moderate sinusoidal space dilatation along with hemorrhages noticed in the sinusoidal space of liver. Few periportal lymphocytes in focal area.

**Fig. 7:**
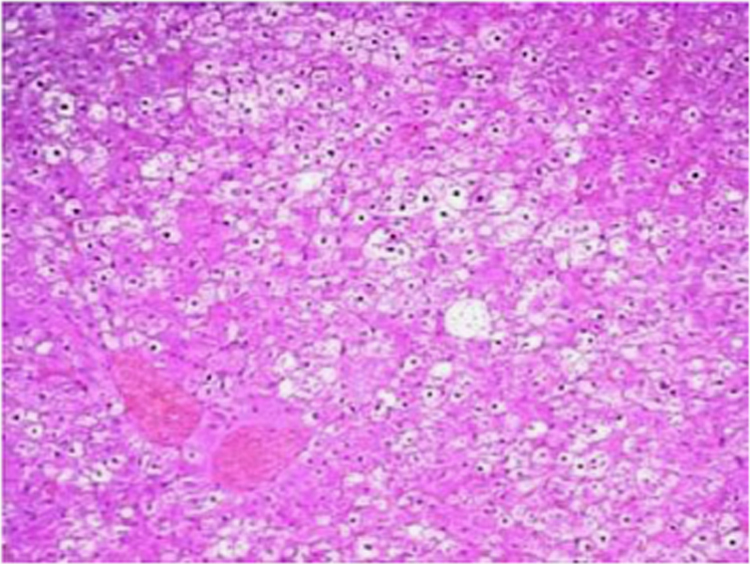
Mild Cord pattern of hepatocytes. Mild sinusoidal space dilation along with hemorrhage. Kupffer cells are normal.

**Fig. 8:**
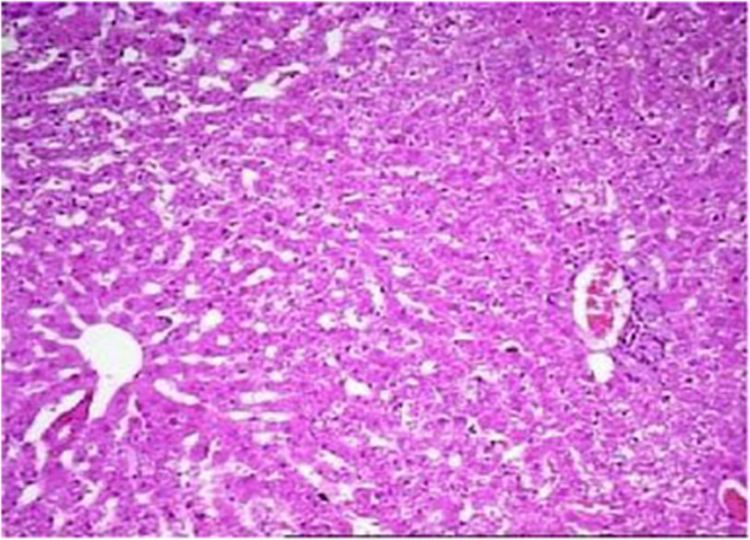
Hepatocytes appeared normal, periportal and centrilobular region appeared normal but mild sinusoidal space dilation along with hemorrhage is noticed in sinusoidal spaces.

**Fig. 9:**
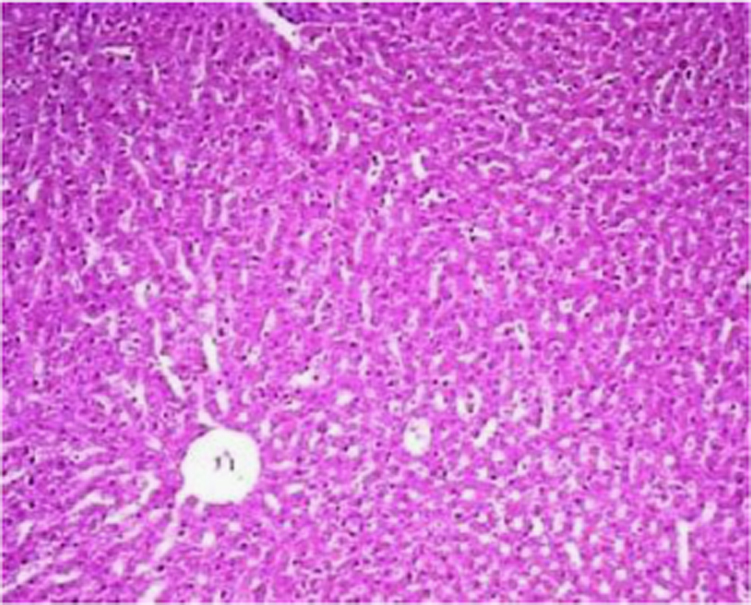
Hepatocytes appeared normal, periportal and centrilobular region appeared normal but mild sinusoidal space dilatation noticed in the pessri portal region of liver.

**Fig. 10:**
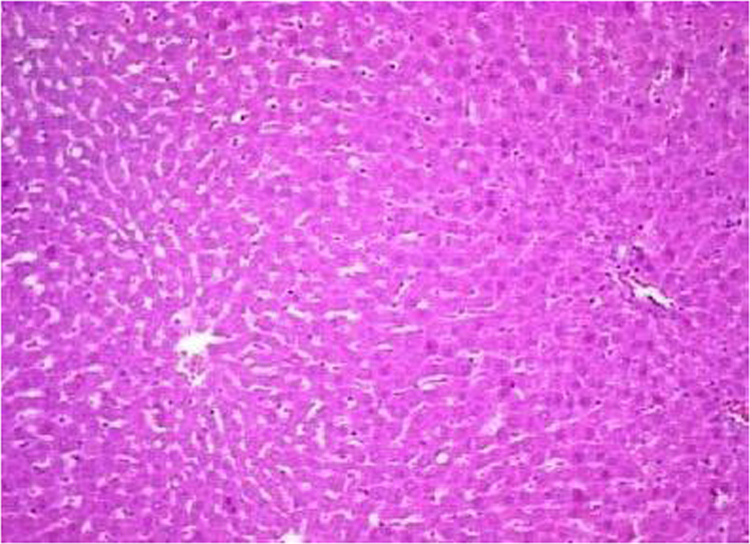
Normal cord pattern of hepatocytes. Periportal few lymphocytes. Kupffer cells and sinusoids appeared to be normal. Periportal few lymphocytes. No evidence of fibrosis.

The present data focuses on antihyperlipidemic capability of *Tagetes patula* Linn. *Tagetes patula* Linn. (French marigold) belongs to the family Asteraceae is widely known for its phytochemical and medicinal properties. The data on chemical composition of methanolic extract *Tagetes patula* Linn was done by gas chromatography and mass spectrometry are shown in Fig. 1 and Table 1. Information regarding changes in lipid profile (TC, TG, LDL, VLDL and HDL) for PUC and triton induced antihyperlipidemic are presented in Table 2 (Fig. 2) and Table 3 (Fig. 3) respectively. Data regarding histological changes of rat hepatocytes of liver are shown in Fig. 4, Fig. 5, Fig. 6, Fig. 7, Fig. 8, Fig. 9, Fig. 10. The present investigation helps in finding the influence of piperine on antihyperlipidemic activity of *Tagetes patula.*

**Table 4:** Antioxidant assay of methanolic flower extract of *Tagetes patula*.

**S.no.**	**Test compounds**	**Antioxidant assay**	**IC**_**50**_**value (µg/mL)**
1	Ascorbic assay (standard)	Hydroxyl radical scavenging assay and Nitric oxide radical scavenging assay	24
2	METP	Hydroxyl radical scavenging assay	38
		Nitric oxide radical scavenging Assay	45

**Table 2:** Anti-hyperlipidemic activity for methanolic extract *Tagetes patula* flower heads on Propylthiouracil induced hyperlipidemic rats.

**Treatment**	**Lipid Profile (mg/dL)**				
	**Total Cholesterol**	**Triglyceride**	**HDL**	**LDL**	**VLDL**
Normal control	161.5±1.36	98.83±2.02	63.33±1.62	80.73±3.13	19.76±0.40
Hyperlipidemic control	258.66±0.9 ^** a^	228.83±1.07^** a^	13.33±0.8^** a^	199.56±1.3^** a^	45.76±0.2^** a^
METP(200 mg/kg)	185.83±1.2 ^** a A^	129.83±0.94 ^** a A^	21.33±1.7^** a B^	138.53±2.1 ^** a A^	25.96±0.1 ^** a A^
METP(200 mg/kg)+piperine(20 mg/kg)	176.83±1.6 ^** a A^	114.33±1.42 ^** a A^	32.16±1.3^** a A^	121.8±2.02^** a A^	22.36±0.2^** a A^
METP(400 mg/kg)	165±1.84^** a A^	101.83±1.27 ^** aA^	41.66±1.3^** a A^	102.96±2.2^** a A^	20.36±0.2^* a A^
METP(400 mg/kg)+piperine(20 mg/kg)	151±1.15^** A^	97.83±0.79^** A^	54.33±1.5^**^	70.6±1.69^* b A^	19.56±0.1^*A^
Simvastatin (10 mg/kg)	155.66±1.1^*A^	93.33±1.33^* A^	57.50±1.0^*A^	78.83±0.83^* A^	18.66±0.2^*A^

Values are expressed as Mean ± SEM, (n=6). Statistical analysis was performed by using ANOVA followed by Dunnett**׳**s test. Results were compared with control group ( ** = p < 0.01, = p < 0.05), hyperlipidemic control (A = p<0.01, B = p<0.05) and standard (a = p < 0.01, b = p < 0.05).

**Table 1:** GC – MS conditions during analysis.

GC CONDITION	**35 °C initial, hold**
Column Oven	
Temperature	time 5 min
Injector	**250°C**
Column Flow	1.2 mL/min
Carrier Gas	Helium 99.9995% Purity
Injection volume	1 mL
MS CONDITION	
Ion source temp	230 °C
MS quard	150 °C
Ionization	EI (-70ev)
Scan speed	2000

## 2 Experimental design, materials and methods

### 2.1 Plant collection and extraction

Flower heads of *Tagetes patula* were procured from plant nurseries in kadiyam, West Godavari district, Andhra Pradesh. Crude material was identified and authenticated by a botanist (Voucher specimen no., TPK-4) from Government Degree College Kukatpally, Hyderabad. The flower heads were dried under shade; coarsely powdered and crude powdered material was used for the extraction process.

### 2.2 Chemicals and reagents

Triton X-100 used was a product of SRL Chemicals, Sisco Research Laboratories PVT LTD. Maharashtra, India. Simvastatin drug used was a product of Sun Pharmaceuticals India LTD., Mumbai, India. Biochemical kits and all other chemicals were of analytical grade.

### 2.3 Preparation of extract

#### 2.3.1 Plant extract

The powdered crude material of *Tagetes patula* was extracted with methanol by Soxhletion and crude extract obtained was evaporated to a solid mass, and preserved in desiccators to remove remaining moisture, if present.

#### 2.3.2 Isolation of piperine

*Piper nigrum* (Black pepper) powder is used for extraction of piperine as per standard methods [1], [2].

### 2.4 Identification of phytochemical constituents using gas chromatography

GC–MS analysis was carried out by Agilent 6890 series GC–MS instrument coupled with mass spectroscopy as a detector. The temperature was adjusted to −30 °C to 280/300 °C. The HP-5MS column with dimensions 30 m ×0.32 mm × 0.25 µm was used for analysis. The oven temperature was adjusted to 35 °C and hold time 5 min, ramp 10 °C / min up to 220 °C. Column flow is 1.2 mL. The inlet temperature was kept at 250 °C and the source temperature of 230 °C and MS Quard temperature of 150 °C (Table 1).

### 2.5 *in vitro* antioxidant assays

The scavenging ability of free radicals as hydroxyl and NO**˙** was measured by the method of Kunchandy and Rao (1990) [3], [4]. Data outcome is shown in Table 4.

### 2.6 Animals

Wistar rats weighing about 170–200 g were procured from Gentox biosciences, Hyderabad for present experimental study. The data protocol was approved by the IAEC (Institutional Animal Ethical Committee Reg. No.1175/PO/ERe/S/08/CPCSEA) of CPCSEA (Committee for control and supervision of experimentation on animals).

### 2.7 Acute toxicity studies

An acute toxicity study up and down procedure (OECD guideline-425) was carried out for methanolic extract of *Tagetes patula* on female Wistar rats [5].

### 2.8 *in vivo* antihyperlipidemic activity of an extract of *Tagetes patula*

#### 2.8.1 Propylthiouracil induced hyperlipidemia

Animals were given with propylthiouracil of 10 mg kg^-1^ *p.o* b. wt. and 0.01% PTU in for 7 days to induce hyperlipidaemia and on 8th day animals are given with test drug orally [6].

The rats were completely randomized into seven groups of six rats each.

Group I: Control (received normal saline).
Group II: Hyperlipidemic rats PTU (10 mg/kg b. wt) 1–8 days + cholesterol (400 mg/kg b. wt) on 8th day.
Group III: PTU (10 mg/kg b. wt) 1–8 days + cholesterol (400 mg/kg b. wt) on 8th day + METP (200 mg/kg b. wt) on 8th day.
Group IV: PTU (10 mg/kg b. wt) 1–8 days + cholesterol (400 mg/kg b. wt) on 8th day + METP (200 mg/kg b. wt) + Piperine (20 mg/kg b. wt) on 8th day.
Group V: PTU (10 mg/kg b. wt) 1–8 days + cholesterol (400 mg/kg b. wt) on 8th day + METP (400 mg/kg b. wt) on 8th day.
Group VI: PTU (10 mg/kg b. wt) 1–8 days + cholesterol (400 mg/kg b. wt) on 8th day + METP (200 mg/kg b. wt)on 8th day + Piperine (20 mg/kg b. wt) on 8th day.
Group VII: Hyperlipidemic rats PTU (10 mg/kg b. wt) 1–8 days + cholesterol (400 mg/kg b. wt) on 8th day+ Simvastatin (10 mg/kg b. wt) on 8th day.

Lipid levels were measured on 8th day using a Cholesterol measurement kit, the data analyzed is presented in Table 2.

#### 2.8.2 Triton induced hyperlipidemic rat model [7]

The rats were completely randomized into seven groups of six rats each.

Group I: Control (received normal saline).
Group II: Triton X-100 (100 mg/kg b. wt *i.p*)
Group III: Triton X-100 (100 mg/kg b. wt *i.p*) + METP (200 mg/kg b. wt).
Group IV: Triton X-100 (100 mg/kg b. wt *i.p*) + METP (200 mg/kg b.wt) + Piperine (20 mg/kg b. wt).
Group V: Triton X-100 (100 mg/kg b. wt *i.p*) + METP (400 mg/kg b. wt).
Group VI: Triton X-100 (100 mg/kg b. wt *i.p*) + METP (400 mg/kg b. wt) + Piperine (20 mg/kg b. wt).
Group VII: Triton X-100 (100 mg/kg b. wt *i.p*) + Simvastatin (10 mg/kg b. wt).

Lipid levels measured using a Cholesterol measurement kit, the data analyzed is presented in Table 3.

**Table 3:** Anti-hyperlipidemic activity for methanolic extract *Tagetes patula* flower heads on Triton induced hyperlipidemic rats.

**Treatment**	**Lipid Profile (mg/dL)**				
	**Total Cholesterol**	**Triglyceride**	**HDL**	**LDL**	**VLDL**
Normal control	163.83±1.81	95.83±1.83	61.16±2.10	83.5±1.91	19.16±0.36
Hyperlipidemic control	260.16±1.7^** a^	225.33±2.09^** a^	15.66±1.2^** a^	199.43±2.06^** a^	45.06±0.4^**a^
METP (200 mg/kg)	187.66±1.42^** a A^	130.5±1.96^** a A^	24.66±2.5^** a B^	136.9±1.69^** a A^	26.1±0.39^** a A^
METP(200 mg/kg)+piperine(20 mg/kg)	181.66±0.95^** a A^	123.5±1.58^** a A^	35±1.50^** a A^	121.96±1.01^** a A^	24.7±0.31^** a A^
MEAB(400 mg/kg)	178.66±1.8 ^** a,A^	115.33±1.60^** a A^	40±1.73^** a A^	112.53±2.98^** a A^	23.06±0.3^** a A^
METP(400 mg/kg)+piperine(20 mg/kg)	172.16±1.6^* b B^	110.83±1.77^** b A^	52±1.34^** b A^	98±1.14^** A b^	19.56±0.1^* A^
Simvastatin (10 mg/kg)	170.5±1.31^* B^	102.83±1.83^** A^	57.83±1.8^* A^	92.1±1.05^* A^	20.56±0.3^* A^

Values are expressed as Mean ± SEM, (n=6). Statistical analysis was performed by using ANOVA followed by Dunnett**׳**s test. Results were compared with control group ( ** = p < 0.01, = p < 0.05), hyperlipidemic control (A = p<0.01, B = p<0.05) and standard (a = p < 0.01, b = p < 0.05).

### 2.9 Histopathology of the liver of propylthiouracil induced diabetic rats

On 8th days of study, the animals were sacrificed to separate livers, which were fixed in 10% formalin for 24 h and used for histopathological studies. The data of histopathological studies were shown in Fig. 4, Fig. 5, Fig. 6, Fig. 7, Fig. 8, Fig. 9, Fig. 10.

### 2.10 Statistical analysis

The results were expressed as mean ± SEM. The results were subjected to statistical analysis by using one way ANOVA followed by Dunnett׳s test p < 0.05, p < 0.01 was considered as statistically significant.
