# Supplementary material for: Data of antihyperlipidaemic activity for methanolic extract of Tagetes patula Linn. flower head along with piperine, as bioavailability enhancer
Source: Data Brief. 2018 Oct 13;21:587–97. doi: 10.1016/j.dib.2018.10.022 (PMC6202789; doi:10.1016/j.dib.2018.10.022)
Supplement: Supplementary file 1 — Supplementary material [file mmc1.docx]

Conflict of Interest and Authorship Conformation Form

Please check the following as appropriate:

- All authors have participated in (a) conception and design, or analysis and interpretation of the data; (b) drafting the article or revising it critically for important intellectual content; and (c) approval of the final version.
- This manuscript has not been submitted to, nor is under review at, another journal or other publishing venue.
- The authors have no affiliation with any organization with a direct or indirect financial interest in the subject matter discussed in the manuscript
- The following authors have affiliations with organizations with direct or indirect financial interest in the subject matter discussed in the manuscript:

Author’s name Affiliation

Dr. Sneha Ramesh Nawale Associate Prof., Department of Pharmacognosy,

Gokaraju Rangaraju College of Pharmacy,

Bachupally, Hyderabad-500090

K. padma priya Student (M.Pharmacy) , Gokaraju Rangaraju College of Pharmacy, Bachupally, Hyderabad-500090.

P. Pranusha Student (M.Pharmacy) , Gokaraju Rangaraju College of Pharmacy, Bachupally, Hyderabad-500090.

Dr. M. Gangaraju Professor and HOD, Department of Pharmacology,

Gokaraju Rangaraju College of Pharmacy, Bachupally, Hyderabad-500090.
