# Supplementary material for: Data of antihyperlipidaemic activity for methanolic extract of Tagetes patula Linn. flower head along with piperine, as bioavailability enhancer
Source: Data Brief. 2018 Oct 13;21:587–97. doi: 10.1016/j.dib.2018.10.022 (PMC6202789; doi:10.1016/j.dib.2018.10.022)
Supplement: Supplementary file 2 — Supplementary material [file mmc2.pdf]

GC CONDITION

Column Oven Temperature	: 35˚C initial, hold time 5min
Injector	250˚C
Column Flow	1.2 mL/min
Carrier Gas	Helium 99.9995% purity
Injection volume	1 mL

MS CONDITION

Ion source temp	230 °C
MS quard	150 °C
Ionization	EI (-70ev)
Scan speed	2000

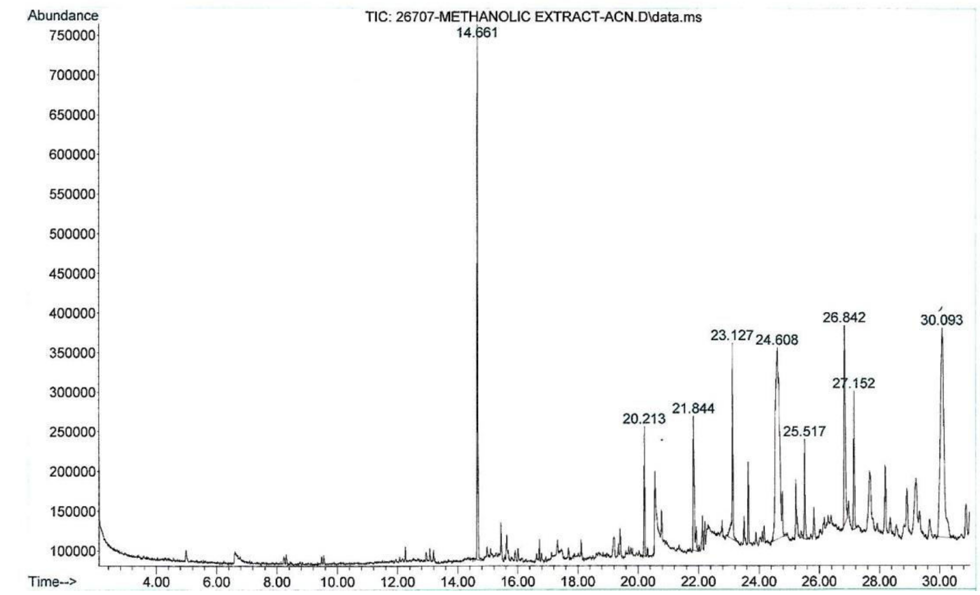

Signal : TIC: 26707-METHANOLIC EXTRACT-ACN.D\data.ms

peak #	R.T. min	first scan	max scan	last scan	PK TY	peak height	corr. area	corr. % max.	% of total
1	14.661	1859	1885	1906	BB	653800	10692366	42.49%	8.409%
2	15.441	1987	2001	2015	BV 7	44723	1015963	4.04%	0.799%
3	15.629	2024	2030	2049	VV 5	31386	1030535	4.10%	0.810%
4	16.722	2155	2193	2199	BV 7	26759	670649	2.67%	0.527%
5	17.322	2219	2283	2291	BV 7	17502	635400	2.53%	0.500%
6	19.409	2591	2596	2611	VV 4	34414	735093	2.92%	0.578%
7	20.213	2699	2716	2724	BV	159652	2607809	10.36%	2.051%
8	20.564	2740	2769	2795	BV 5	96436	4858765	19.31%	3.821%
9	20.777	2795	2801	2826	VB 2	46870	1583221	6.29%	1.245%
10	21.844	2910	2961	2984	BV	164703	5145387	20.45%	4.046%
11	22.130	2984	3003	3011	PV 4	41977	969838	3.85%	0.763%
12	22.211	3011	3015	3020	PV 2	33326	548420	2.18%	0.431%
13	22.320	3020	3032	3061	VB 2	23000	2092979	8.32%	1.646%
14	23.127	3111	3153	3187	BB	240128	6330584	25.16%	4.978%
15	23.508	3196	3210	3221	BV 3	36727	882700	3.51%	0.694%
16	23.647	3221	3231	3252	VV	103088	1920432	7.63%	1.510%
17	24.608	3329	3375	3437	BB 3	242730	25164196	100.00%	19.790%
18	25.223	3438	3467	3487	BV 8	71322	2065605	8.21%	1.624%
19	25.517	3503	3511	3523	BB	120359	2232459	8.87%	1.756%
20	25.823	3537	3557	3567	BV 4	39021	1081556	4.30%	0.851%
21	26.842	3689	3709	3723	BV 2	248588	7838684	31.15%	6.164%
22	26.968	3723	3728	3748	VV 4	37478	1454031	5.78%	1.143%
23	27.152	3748	3756	3786	VB	170284	4045563	16.08%	3.181%
24	27.678	3787	3835	3861	BV	74932	4920646	19.55%	3.870%
25	28.194	3887	3912	3929	BV	84077	2339845	9.30%	1.840%
26	28.916	3989	4020	4042	BV 6	62357	3533582	14.04%	2.779%
27	29.215	4042	4065	4077	VV 6	74520	5172765	20.56%	4.068%
28	29.335	4077	4083	4107	VB 9	32745	1563249	6.21%	1.229%
29	30.093	4159	4196	4269	BB 3	264727	22614602	89.87%	17.784%
30	30.886	4304	4315	4325	PV 2	39915	1412276	5.61%	1.111%

Fig. 1: Gas chromatogram and mass spectrometry spectra of methyl extract of flower heads of *Tagetes patula* (METP).
