# Supplementary material for: Data of antihyperlipidaemic activity for methanolic extract of Tagetes patula Linn. flower head along with piperine, as bioavailability enhancer
Source: Data Brief. 2018 Oct 13;21:587–97. doi: 10.1016/j.dib.2018.10.022 (PMC6202789; doi:10.1016/j.dib.2018.10.022)
Supplement: Supplementary file 3 — Supplementary material [file mmc3.pdf]

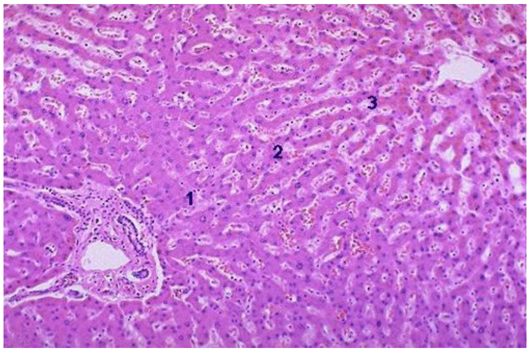

A

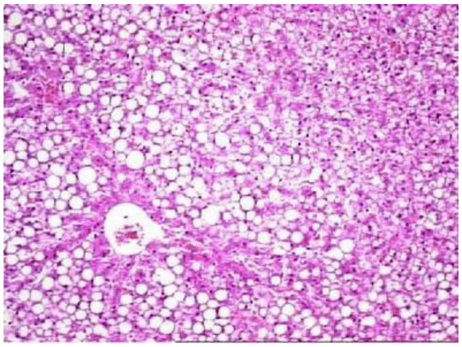

B

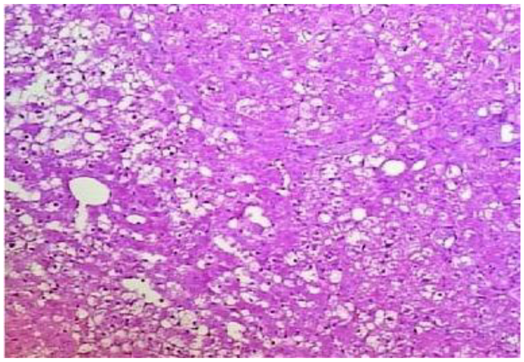

C

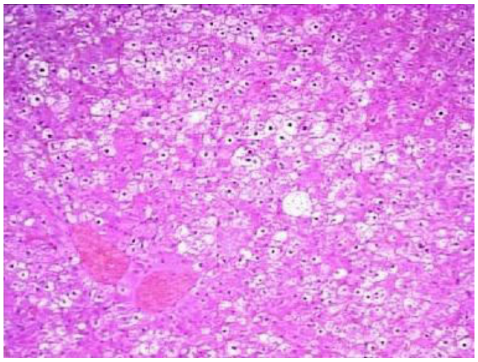

D

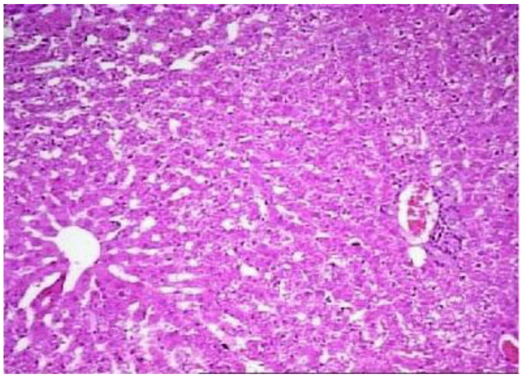

E

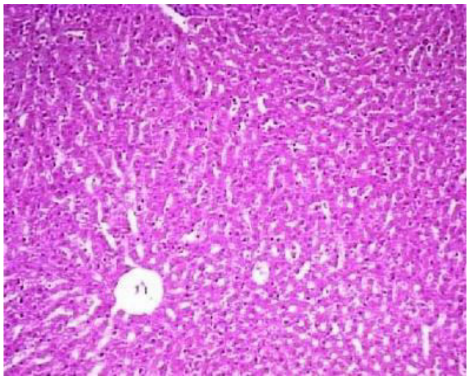

F

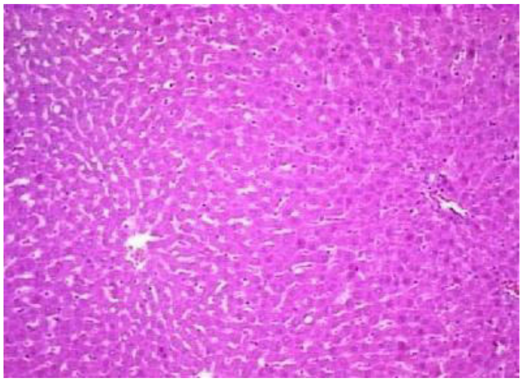

G

Fig 4: Histological changes of rat liver

A) Histopathology of rat's liver in control group, Bile duct appeared normal, no inflammation or fibrosis noticed surrounding the portal region of liver. Kupffer cells and sinusoids are normal. No evidence of fatty change and fibrosis. B) Cord pattern of hepatocytes. Few periportal lymphocytes in focal area. fibrosis noticed in periportal region of liver. Fatty change found in cytoplasm and fibrosis. C) Moderate sinusoidal space dilatation along with hemorrhages noticed in the sinusoidal space of liver. Few periportal lymphocytes in focal area. D) Mild Cord pattern of hepatocytes. Mild sinusoidal space dilation along with hemorrhage. Kupffer cells are normal. E) Hepatocytes appeared normal, periportal and centrilobular region appeared normal but mild sinusoidal space dilation along with hemorrhage is noticed in sinusoidal spaces. F) Hepatocytes appeared normal, periportal and centrilobular region appeared normal but mild sinusoidal space dilatation noticed in the pessri portal region of liver. G) Normal cord pattern of hepatocytes. Periportal few lymphocytes. Kupffer cells and sinusoids appeared to be normal. Periportal few lymphocytes. No evidence of fibrosis.
